# Anti-TLR7 Antibody Protects Against Lupus Nephritis in NZBWF1 Mice by Targeting B Cells and Patrolling Monocytes

**DOI:** 10.3389/fimmu.2021.777197

**Published:** 2021-11-11

**Authors:** Yusuke Murakami, Ryutaro Fukui, Reika Tanaka, Yuji Motoi, Atsuo Kanno, Ryota Sato, Kiyoshi Yamaguchi, Hirofumi Amano, Yoichi Furukawa, Hitoshi Suzuki, Yusuke Suzuki, Naoto Tamura, Naomi Yamashita, Kensuke Miyake

**Affiliations:** ^1^ Division of Innate Immunity, Department of Microbiology and Immunology, The Institute of Medical Science, The University of Tokyo, Tokyo, Japan; ^2^ Faculty of Pharmacy, Department of Pharmaceutical Sciences & Research Institute of Pharmaceutical Sciences, Musashino University, Tokyo, Japan; ^3^ Division of Clinical Genome Research, Advanced Clinical Research Center, The Institute of Medical Science, The University of Tokyo, Tokyo, Japan; ^4^ Department of Internal Medicine and Rheumatology, Juntendo University School of Medicine, Tokyo, Japan; ^5^ Department of Nephrology, Juntendo University Faculty of Medicine, Tokyo, Japan; ^6^ Laboratory of Innate Immunity, Center for Experimental Medicine and Systems Biology, The Institute of Medical Science, The University of Tokyo, Tokyo, Japan

**Keywords:** toll-like receptor, lupus nephritis, inhibitory monoclonal antibody, monocytes, autoantibody

## Abstract

Systemic lupus erythematosus (SLE) is an autoimmune disease characterized by autoantibody production and multiple organ damage. Toll-like receptor 7 (TLR7), an innate immune RNA sensor expressed in monocytes/macrophages, dendritic cells (DCs), and B cells, promotes disease progression. However, little is known about the cellular mechanisms through which TLR7 drives lupus nephritis. Here, we show that the anti-mouse TLR7 mAb, but not anti-TLR9 mAb, protected lupus-prone NZBWF1 mice from nephritis. The anti-TLR7 mAb reduced IgG deposition in glomeruli by inhibiting the production of autoantibodies to the RNA-associated antigens. We found a disease-associated increase in Ly6C^low^ patrolling monocytes that expressed high levels of TLR7 and had upregulated expression of lupus-associated IL-10, CD115, CD31, and TNFSF15 in NZBWF1 mice. Anti-TLR7 mAb abolished this lupus-associated increase in patrolling monocytes in the circulation, spleen, and glomeruli. These results suggested that TLR7 drives autoantibody production and lupus-associated monocytosis in NZBWF1 mice and, that anti-TLR7 mAb is a promising therapeutic tool targeting B cells and monocytes/macrophages.

## Introduction

Systemic lupus erythematosus (SLE) is an autoimmune disease of unknown etiology characterized by autoantibody production and clinical manifestations affecting the skin, joints, kidneys, and central nervous system (1). Immunosuppressive agents such as antimalarial drugs, hydroxychloroquine (HCQ), non-steroidal anti-inflammatory drugs, glucocorticoids, and mycophenolate mofetil have been administered to control SLE. However, life-threatening manifestations, such as lupus nephritis, develop in resistant patients despite such treatments. Furthermore, the use of glucocorticoids is limited due to various adverse effects. Therefore, a novel therapeutic agent with fewer adverse effects is required.

Causative autoimmune responses are driven by autoreactive B cells that produce autoantibodies to nucleic acid (NA)-associated autoantigens, conventional dendritic cells (cDCs) that produce proinflammatory cytokines, and plasmacytoid dendritic cells (pDCs) that produce type I interferons (IFNs) (2, 3). In addition to these cells, monocytes/macrophages infiltrate glomeruli and play pathogenic roles in glomerular damage associated with SLE, independently of immune complex deposition (4–6).

Toll-like receptor 7 (TLR7) is an innate immune RNA sensor that is expressed in B cells, dendritic cells, and monocytes/macrophages. This receptor responds not only to pathogen-derived single-stranded RNA (ssRNA), but also to self-derived ssRNA, and drives autoimmune diseases such as SLE and psoriasis (7–9). A lupus-prone mouse strain, Y-linked autoimmune accelerator (Yaa), has a duplicate copy of the TLR7 gene that results in TLR7 hyperactivation, leading to lupus-like states (10, 11). The TLR7 agonist imiquimod drives lupus nephritis in mice (12, 13), whereas lupus nephritis spontaneously developed in the lupus-prone strain, New Zealand Black/New Zealand White F1 mice (NZBWF1 mice) is ameliorated by a small chemical TLR7 inhibitor (14). The immune complex (IC)-independent glomerular accumulation of Ly6C^low^ patrolling monocytes causes lupus nephritis in lupus-prone mouse strain lacking the human SLE susceptibility gene *Tnip1* (6). Although both TLR7 and TLR9 drive lupus nephritis in this strain (6), TLR7 might play unique pathogenic roles in patrolling monocytes because they express abundant TLR7 (15).

We previously reported that the anti-TLR7 mAb inhibits TLR7 responses in B cells, dendritic cells, and monocyte/macrophages (16). The anti-TLR7 mAb binds to cell surface TLR7, which is internalized into the endosomal compartment. Because TLR7 shuttles between cell surface and the endosomal compartment, endosomal TLR7 comes out of the cell surface and becomes accessible to the anti-TLR7 mAb (16). Therefore, the TLR7-mAb immune complex gradually increases with the mAb treatment. When endosomal TLR7 is mostly complexed with the anti-TLR7 mAb, endosomal TLR7 responses are inhibited. The inhibitory effect of the anti-TLR7 was also observed *in vivo*, rescuing *Unc93b1* ^D34A/D34A^ mice from TLR7-dependent autoimmune hepatitis. Here, we investigated the pathogenic role of TLR7 in NZBWF1 mice using an anti-TLR7 inhibitory mAb This mAb ameliorated lupus nephritis in NZBWF1 mice by acting on B cells and monocytes/macrophages, thereby reducing IgG deposition in glomeruli and diminishing autoantibody production. These findings suggested that the activation and differentiation of autoreactive B cells in NZBWF1 mice is TLR7-dependent. Furthermore, the numbers of Ly6C^low^ patrolling monocytes, which are thought to be tissue macrophages in the circulation, TLR7-dependently increased in the spleen, circulation, and kidneys. Transcriptome and FACS analyses revealed increased expression of lupus-associated molecules such as IL-10, which promotes nephritis, in monocytes that accumulated in the spleen (17). These results suggested that TLR7 is a therapeutic target for SLE and that anti-TLR7 mAb is a promising therapeutic tool targeting both B cells and monocytes in SLE.

## Material and Methods

### Reagents and Antibodies

Standard saline was purchased from Otsuka Pharmaceutical Co., Ltd. (Tokushima, Japan). Anti-mouse TLR7 mAb (mouse IgG1, κ, clone A94B10), anti-mouse TLR9 mAb (mouse IgGa, κ, clone NaR9), and isotype control mAb (IgG1, κ, clone TF904 or IgG2a, κ, clone YN907) were purified by us. FITC-conjugated anti-mouse IgG antibody was purchased from Southern Biotech (1030-02, Birmingham, UK). FITC-conjugated anti-mouse C3 antibody was purchased from MP Biomedicals (SKU:0855500, CA, USA). The anti-mouse CD19 (clone 6D5), GL7 (clone GL7), CD8α (clone 53-6.7), CD11c (clone N418), Ly-6C (clone HK1.4), Ly-6G (clone 1A8), TREML4 (clone 16E5), CD273 (clone TY25), CD31 (clone 390), CD115 (clone AFS98), CD14 (clone Sa14-2), CD41 (clone MWReg30), CD85k (clone H1.1), CD54 (clone YN1/1.7.4), CD132 (clone TUGm2), PD-1H (clone MH5A), CD169 (clone 3D6.112), CD274 (clone 10F.9G2), CD61 (clone 2C9.G2), integrin β7 (clone FIB27), CD63 (clone NVG-2), CD88 (clone 20/70), TER119 (clone TER-119), CD117 (2B8), and NK1.1 (clone PK136) antibodies were purchased from BioLegend (San Diego, CA, USA). Anti-mouse CD138 (clone 281-2), I-A/I-E (clone M5/114), CD3ϵ (clone 145-2C11), CD4 (clone GK1.5), CD44 (clone IM7), CD62L (clone MEL-14), CD49b (clone HMα2), CD11b (clone M1/70), Siglec-H (clone 440c), CD16.2 (clone 9E9), and CD45.2 (clone 104) antibodies were purchased from BD (Franklin Lakes, NJ, USA). Staining buffer (1x PBS, 2.5% FBS, 2 mM EDTA, and 0.1% sodium azide) was prepared by us. The LEGENDScreen Mouse PE Kit was purchased from BioLegend.

### Mice

Female C57BL/6NCrSlc and NZBWF1/Slc mice were purchased from Japan SLC, Inc. (Shizuoka, Japan). The mice were housed in a specific-pathogen-free (SPF) environment with free access to food and water, under the approval of the Animal Experiment Committee of The Institute of Medical Science, The University of Tokyo (approval numbers PA-84 and A17-83).

### Antibody Treatment

10 mg/kg of anti-mouse TLR7 mAb, isotype control mAb, or the same volume of saline was injected into the peritoneal cavity of NZBWF1 mice once a week. Administration was started at 12-16 weeks of age and ended at 35-40 weeks.

### Biochemical Tests

Urine and serum were collected from mice aged 30-40 weeks. Urine albumin, urine creatinine, and blood urea nitrogen (BUN) levels were measured by ORIENTAL YEAST CO., LTD. (Tokyo, Japan).

### Histological Analysis

Mouse kidneys were fixed in 20% formalin neutral buffer solution. Fixed kidneys were then embedded in paraffin wax for sectioning. The sections were stained with hematoxylin and eosin (HE) or periodic acid-Schiff (PAS). The sections were visualized using an EVOS microscope (Thermo Fisher Scientific, Waltham, Massachusetts, USA). Pathological scores for glomerulonephritis were defined as the average of the scores derived from 50 glomeruli. Glomerulonephritis was scored as 0, normal; 1, cell proliferation or infiltration; 2, membranoproliferation, lobulation, or hyaline deposition and 3, crescent formation or global hyalinosis.

### Immunohistochemistry (IHC)

Mouse kidneys were embedded in Tissue Tek embedding medium for frozen tissue blocks (Sakura Finetechnical Co., Ltd., Tokyo, Japan). Frozen kidney sections were sectioned and incubated with FITC-conjugated anti-mouse IgG antibody (Southern Biotech Birmingham, UK). Stained samples were mounted with Fluoromount/Plus (Diagnosis Biosystems, CA, USA). All samples were visualized using an FM1000D confocal laser scanning microscope (Olympus, Tokyo, Japan), and the images were analyzed using the FV10-ASW viewer (Olympus) or Image J software (Schneider, Nat. Methods 2012). For the IHC of color development, frozen kidney sections were incubated with anti-mouse CD11b (clone M1/70), anti-mouse CD16.2 (FcγRIV) (clone 9E9), anti-mouse TREML4 (clone 16E5), and anti-mouse Ly-6G (clone 1A8). Samples were mounted with Fluoromount/Plus (Diagnosis Biosystems) and analyzed using an EVOS microscope (Thermo Fisher Scientific) or a BZ-X710 fluorescence microscope (Keyence, Osaka, Japan). The number of glomeruli assessed was 5 to 10 from an individual kidney in B6 WT (*n* = 4 or 5), saline (*n* = 5), control IgG1 (*n* = 6), and anti-TLR7 (*n* = 5). The dots show the average percentage of the indicated positive staining area. Ratios (%) of the indicated positive staining area to the total area of one glomerulus were calculated using the BZ-X710 fluorescence microscope software.

### ELISA

Anti-Sm and anti-SSA/Ro60 antibodies were measured using an ELISA kit (Alpha Diagnostic International Inc., San Antonio, TX, USA). Anti-double-stranded DNA antibodies in serum were measured using an ELISA kit (FUJIFILM Wako Pure Chemical Corporation, Osaka, Japan). Serum IL-10 was measured using a Duo Set ELISA kit (R&D Systems, Minneapolis, MN, USA). Serum ACP5 levels were measured using an ELISA kit (Novus Biologicals, MN, USA).

### Preparation of Cells in Tissues and Peripheral Blood

Spleens were harvested from mice and dissociated into single-cell suspensions with glass slides. Single cell suspensions of kidney were prepared using Multi Tissue Dissociation Kit 2 and Debris Removal Solution (Miltenyi Biotec, Bergisch Gladbach, Germany). Peripheral blood was harvested from the buccal blood vessels and kept in EDTA-treated microtubes. Red blood cells in the prepared samples were lysed using RBC Lysis Buffer (BioLegend).

### Flow Cytometry Analysis

Fc receptors on the cells were blocked by non-conjugated anti-CD16/32 (2.4G2, Bio X cell, Lebanon, NH, USA) for 10 min at room temperature. Cells were stained with antibodies for 15 min at 4°C. Stained cells were fixed with BD Cytofix Fixation Buffer (BD) for 20 min at 4°C and washed twice with staining buffer 2 times. For intracellular staining of TLRs, fixed cells were permeabilized using BD Perm/Wash buffer (BD) and incubated with anti-TLR antibody for 30 min at 4°C. Prepared cells were suspended in staining buffer and analyzed by BD FACS Aria III, BD LSR Fortessa X-20, or BD FACSLyric. The obtained data were analyzed using the FlowJo software (BD). Antibodies were diluted with a staining buffer. The reaction of these steps was performed at 1 × 10^7^ cells/mL.

### RNA Extraction and cDNA Synthesis

RNA was prepared from cells using the RNeasy Mini or Micro kit (Qiagen, Venlo, Netherlands). To extract RNA from the kidneys, the kidneys were incubated with Sepasol-RNA I Super G solution for RNA isolation (Nacalai Tesque, Osaka, Japan) and homogenized with metal beads in a multi-bead shocker (Yasui Kikai, Osaka, Japan). Chloroform (200 μL/kidney) was added and centrifuged at 15300 × *g* at 4°C for 15 min. The supernatant was collected, and 500 μL/kidney isopropanol was added. The sample was mixed well, and the mixture was centrifuged at 15300 × *g* at 4°C for 10 min. The supernatant was discarded, and 70% ethanol was added to the nucleic acid pellets. The pellet was then centrifuged at 15300 g at 4°C for 5 min. The supernatant was removed, and the nucleic acid pellet was dried and dissolved in 200 μL of water. Complementary DNA (cDNA) was synthesized from the extracted RNA using ReverTra Ace qPCR Master Mix (Toyobo, Osaka, Japan).

### Real-Time PCR

To measure mRNA levels, cDNA was quantified by real-time PCR using TaqMan probes and primers (Thermo Fisher Scientific) in a total volume of 20 μL. mRNA expression was calculated according to the comparative threshold cycle method, using the hypoxanthine-guanine phosphoribosyltransferase gene (*Hprt*) as an internal control. The accession numbers of TaqMan probes are; *Hprt*: Mm03024075_m1, *Tnfsf15*: Mm00770031_m1, *Il10*: Mm01288386_m1, *Acp5*: Mm00475698_m1, *Il34*: Mm01243248_m1, *Csf1*: Mm00432686_m1, *Tnf*: Mm00443258_m1, *Il6*: Mm00446190_m1, *Il12b*: Mm01288989_m1, *Cxcl1*: Mm04207460_m1, *Ifna4*: Mm00833969_s1, *Ifnb1*: Mm00439552_s1.

### Cell Sorting

Single-cell suspensions of splenocytes were prepared with 0.65 WU/ml of Liberase TL and 200 U/ml of DNase I (Sigma-Aldrich, St. Louis, MO, USA). After RBC lysis, Fc receptors on the cells were blocked, as previously described. To exclude T cells, B cells, NK cells, erythroblasts, granulocytes, and cDCs, cells were incubated with biotin-conjugated anti-mouse CD3ϵ, CD19, NK1.1, TER119, Ly6G, and CD117. Labeled cells were incubated with anti-biotin MACS beads (Miltenyi Biotec), and crude monocytes were collected as the negative fraction using Auto MACS separator (Miltenyi Biotec). Cells were stained with anti-mouse CD11b, CD11c, Ly6C, I-A/I-E, CD49b, and CD16.2 as described. Stained cells were subjected to cell sorting with BD FACS Aria III to collect patrolling monocytes and classical monocytes (**Figure 3E**). Collected cells were suspended in RNA protect cell reagent (QIAGEN) and stored at -80°C.

### RNA Sequencing

Total RNA was extracted from the cells as previously described. RNA samples were checked using a Bioanalyzer 2100 with RNA 6000 nano kit (Agilent Technologies, Santa Clara, CA, USA), and RNA integrity number (RIN) was calculated. The high-quality RNA (RIN > 7.2) samples were subjected to RNA sequencing using the Ion Torrent NGS system (Thermo Fisher Scientific). Briefly, RNA libraries were prepared using 10.62 ng of total RNA with an Ion AmpliSeq Transcriptome Mouse Gene Expression kit, and sequenced on Ion Proton using an Ion PI Hi-Q Sequencing 200 kit and Ion PI Chip v3 (Thermo Fisher Scientific). The data were analyzed using AmpliSeqRNA plug-in v5.2.0.3 in the Torrent Suite Software v5.2.2 (Thermo Fisher Scientific), and normalized using RPM (reads per million mapped reads) method. The normalized data were further analyzed and visualized using GeneSpring v14.9.1 software (Agilent Technologies), R Studio (R Foundation for Statistical Computing, Vienna, Austria), and Microsoft Excel (Microsoft, Redmond, WA, USA).

### Statistics

Statistical significance was calculated by performing Log-rank test, two-tailed unpaired Student’s *t*-test, Welch’s *t*-test, one-way ANOVA, or two-way ANOVA. A *p-*value of < 0.05 was considered statistically significant. If the result of one-way ANOVA was significant (*p* < 0.05), Tukey’s multiple comparison test was performed. Prism software (GraphPad Software, San Diego, CA, USA) was used for the statistical analyses.

## Results

### Anti-TLR7 mAb Protected NZBWF1 Mice From Lupus Nephritis

**Figure 1:**
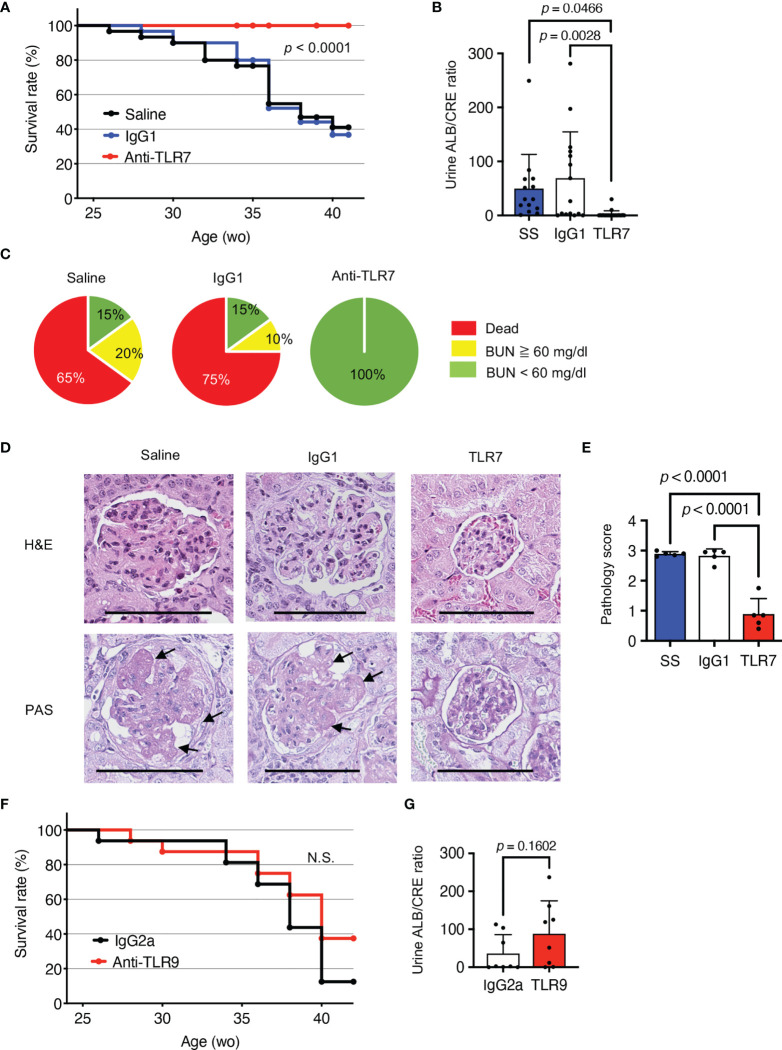
Protective effects of anti-TLR7 mAb against lupus nephritis. **(A-E)** NZBWF1 mice were intraperitoneally administered 10 mg/kg of anti-TLR7 mAb (clone A94B10, IgG1), isotype control (clone TF904, IgG1) mAb or standard saline (control) weekly from age 12-16, until 35-40 weeks. **(A)** Survival of NZBWF1 mice (*n* = 30 per group). **(B)** Urinary albumin (ALB)/creatinine (CRE) ratio in 30-40-week-old NZBWF1 mice (*n* = 30). **(C)** Ratios (%) of mice with blood urea nitrogen (BUN) above (yellow) or below (green) 60 mg/dL at age of 40 weeks and those of dead mice (red; *n* = 20). **(D)** Representative images of glomerular sections visualized by hematoxylin and eosin (HE) or periodic acid-Schiff (PAS) staining. Arrows, increased numbers of mesangial cells. Scale bar, 100 μm. **(E)** Pathology scores from 0 to 3 (*n* = 5). **(F, G)** NZBWF1 mice were administered anti-TLR9 mAb (clone NaR9, IgG2a, *n* = 16) or isotype control mAb (clone YN907, IgG2a, *n* = 16) at 10 mg/kg from age 12-40 weeks. **(F)** Survival curves of mice administered Ab (*n* = 16). **(G)** Urinary ALB/CREA ratio in mice aged 30-40 weeks (*n* = 8). Data were statistically analyzed using Log-rank tests **(A, F)**, one-way ANOVA with Tukey’s multiple comparison tests **(B, E)**, or Student’s *t*-tests **(G)**. Data are shown as individual points and as means ± SD for each experimental group **(B, E, G)**.

To understand the role of TLR7 in disease progression in NZBWF1 mice, we intraperitoneally administered anti-TLR7 mAb (10 mg/kg weekly) to 12-16-week-old NZBWF1 mice. By the age of 40 weeks, all mice that were administered the anti-TLR7 mAb remained alive, whereas 60%-75% of the mice administered saline or the isotype control Ab died (**Figure 1A**). The NZBWF1 mice died of kidney failure, indicated by elevated urinary albumin/creatinine (ALB/CRE) ratios and blood urea nitrogen (BUN) (**Figures 1B, C**). These values did not increase in mice administered the anti-TLR7 mAb. Consistent with these findings, histological analyses revealed glomerular changes such as mesangial cell proliferation in mice administered control IgG1 or saline, but significantly less changes in those administered the anti-TLR7 mAb (**Figures 1D, E**). These results suggested that the anti-TLR7 mAb protected the NZBWF1 mice from lupus nephritis.

The single-stranded DNA (ssDNA) sensor, TLR9, also promotes lupus nephritis in lupus-prone *Tnip1* ^-/-^ mice (6). To assess the role of TLR9 in lupus nephritis, we administered our inhibitory anti-TLR9 mAb to NZBWF1 mice (18). Although the anti-TLR9 mAb ameliorates TLR9-dependent lethal hepatitis (18), we did not identify any healing effect in NZBWF1 mice (**Figures 1F, G**). These results suggested that TLR9 is dispensable for disease progression in NZBWF1 mice.

### Anti-TLR7 mAb Reduced IgG Deposition in Glomeruli and Autoantibody Production

**Figure 2:**
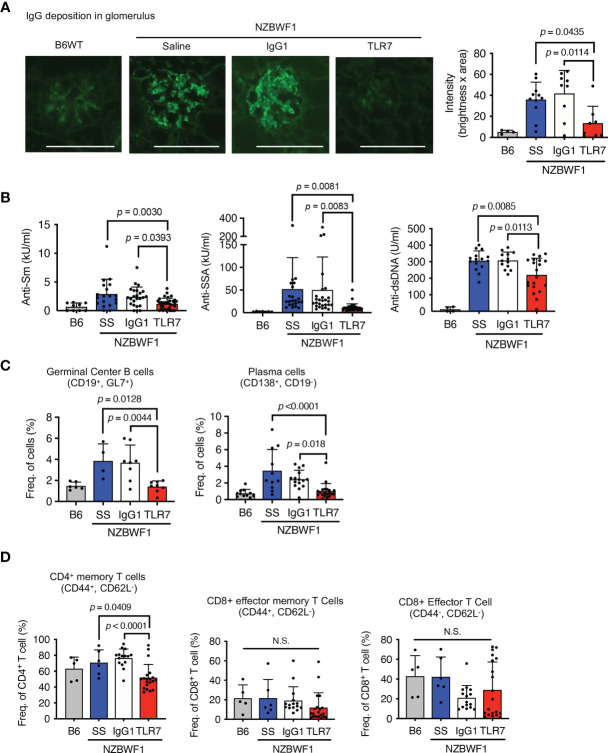
Inhibition of IgG deposition in glomeruli and autoantibody production induced by anti-TLR7 mAb. **(A–D)** NZBWF1 mice were administered saline, IgG1 or anti-TLR7 mAb from ages of 12-16 to 35-40 weeks and compared with age-matched WT C57BL/6 (B6) mice. **(A)** Representative images of immunohistological staining with anti-IgG Ab (left). Scale bar, 100 μm. Quantitation of fluorescence intensity in IgG-positive areas in glomeruli (right). Numbers of glomeruli assessed: B6 (*n* = 4), saline (*n* = 9), control IgG1 (*n* = 9), and anti-TLR7 (*n* = 7). **(B)** Serum levels of autoantibodies in mice aged 30-40 weeks. B6 (*n* ≥ 4), saline (*n* ≥ 15), control IgG1 (*n* ≥ 13), and anti-TLR7 mAb (*n* ≥ 20). **(C, D)** Ratios of B **(C)** and T **(D)** cell subsets in spleen. B6 (*n* ≥ 4), saline (*n* ≥ 4), control IgG1 (*n* ≥ 8), and anti-TLR7 mAb (*n* ≥ 8). Data were statistically analyzed using one-way ANOVA with Tukey’s multiple comparison tests. N.S., not significant. Data are shown as individual points and as means ± SD for each experimental group.

The anti-TLR7 mAb significantly decreased IgG deposition in glomeruli (**Figure 2A**). A previous study found that TLR7 drives autoantibody production in response to RNA-associated autoantigens in MRL/lpr mice (7). In NZBWF1 mice, autoantibodies to Sm and SSA antigens were detectable at 20 wk old and their titer increased with age (**Supplementary Figure 1** and **Figure 2B**). The anti-TLR7 mAb significantly reduced the titers of these se autoantibodies to Sm and SSA antigens at 30-40 wk old. Anti-TLR7 mAb weakly but significantly decreased serum levels of anti-dsDNA autoantibodies (**Figure 2B**). Although production of anti-dsDNA autoantibodies depends on TLR9, not TLR7, in MRL/lpr mice (7), TLR7 partially contributes to production of anti-dsDNA autoantibodies in TLR7 transgenic mice (19). Because TLR7 responds to DNA-derived deoxyguanosine (20), TLR7, in addition to TLR9, would be activated by DNAs in autoreactive B cells. TLR7 activation by DNA would drive anti-dsDNA autoantibody production. The anti-TLR7 mAb would directly act on autoreactive B cells, because it inhibits B cell responses to TLR7 ligands *in vitro* (16).

We analyzed splenic B cell subsets by flow cytometry to assess the effects of the anti-TLR7 mAb on B cells in NZBWF1 mice. The anti-TLR7 mAb decreased the higher ratios (%) of germinal center B cells and plasma cells, in NZBWF1, compared to those in C57BL/6 mice (**Figure 2C**). The mAb did not alter the frequencies of naïve and memory B cells (**Supplementary Figure 2**). These results suggested that the anti-TLR7 mAb inhibits the TLR7-dependent activation and differentiation of autoreactive B cells, leading to reduced levels of serum autoantibodies and decreased IgG deposition in the glomeruli.

The anti-TLR7 mAb also significantly decreased ratios (%) of CD4^+^ memory T cells, but not those of CD8^+^ T cells (**Figure 2D**). Because the anti-TLR7 mAb inhibited TLR7 responses in dendritic cells (DCs) *in vitro* (16), its inhibitory effect on DCs might decrease CD4^+^ memory T cells in NZBWF1 mice.

### Increases of Patrolling Monocytes Were Inhibited by the Anti-TLR7 mAb

**Figure 3:**
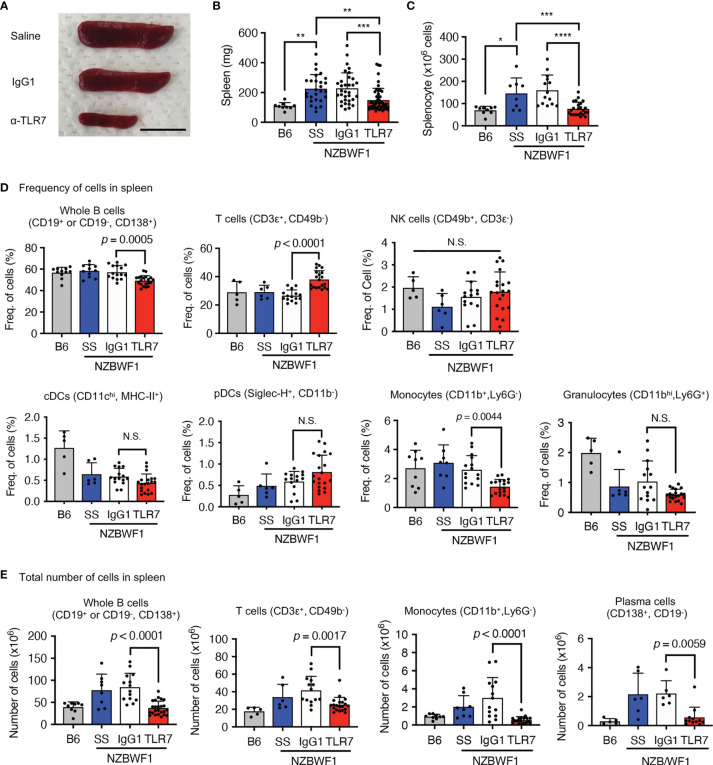
Anti-TLR7 mAb inhibited monocytosis in spleens. **(A-D)** Age-matched B6 and NZBWF1 mice were administered saline, IgG1 or anti-TLR7 mAb from age of 12-16 to 35-40 weeks. **(A-C)** Macroscopic appearance **(A)**, weight **(B)**, and cell numbers **(C)** in spleens. B6 (*n* = 9), saline (*n* = 8), control IgG1 (*n* ≥ 14), and anti-TLR7 mAb (*n* ≥ 24). **(D, E)** Ratios **(D)** and absolute numbers **(E)** of indicated immune cells in spleens: B6 (*n* ≥ 5), saline (*n* ≥ 6), control IgG1 (*n* ≥ 15), and anti-TLR7 (*n* ≥ 18). Data were statistically analyzed using one-way ANOVA. The results found significant by ANOVA (*p* < 0.05) were further assessed by Tukey’s multiple comparison tests **(B, C)**. For flow cytometry analysis, Tukey’s multiple comparison test was performed between control IgG1 and anti-TLR7 groups, and the *p*-values are shown **(D, E)**. *p < 0.05, **p < 0.01, ***p < 0.001, **** *p* < 0.0001. N.S., not significant. Data are shown as individual points and as means ± SD for each experimental group.

**Figure 4:**
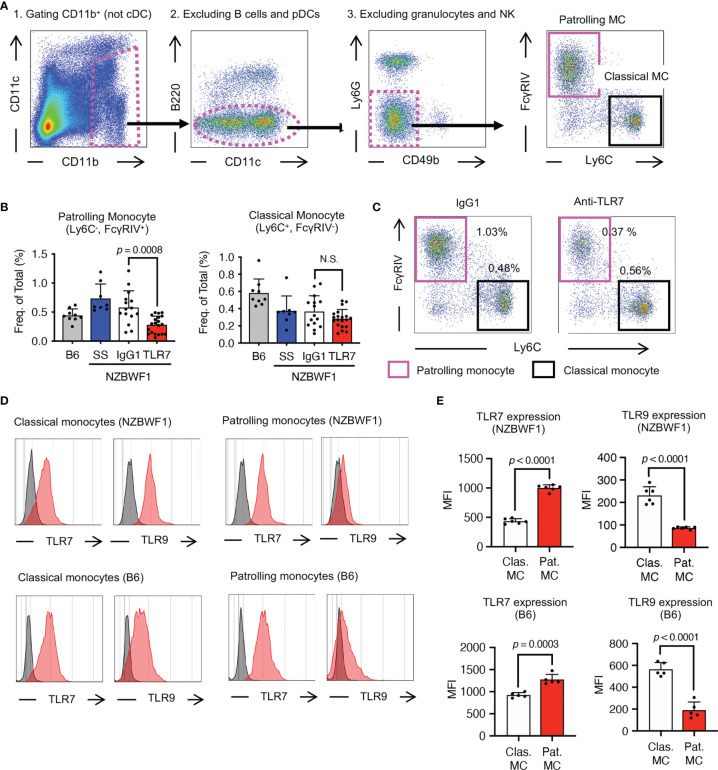
Patrolling monocytes TLR7-dependently increased in NZBWF1 mice. **(A)** Gating strategy and markers for flow cytometry of monocyte subsets. Cells in magenta dotted gates were assessed using drill-down analyses. **(B, C)** NZBWF1 mice were administered saline, IgG1 or the anti-TLR7 mAb from age 12-16 to 35-40 weeks and compared with age-matched B6 mice **(B)** Ratios of monocyte subsets in spleens: B6 (*n* ≥ 5), saline (*n* ≥ 6), control IgG1 (*n* ≥ 15), and anti-TLR7 (*n* ≥ 18). **(C)** Representative dot plots show monocyte subsets in NZBWF1 mice administered IgG1 or anti-TLR7 mAb. **(D)** Red histograms show expression of TLR7 and TLR9 in classical and patrolling monocytes in spleens of 14-week-old NZBWF1 mice detected by membrane permeabilized staining. Gray histograms, staining with isotype-matched control antibodies. **(E)** Statistical analysis of mean fluorescence intensity (MFI) of TLR7 and TLR9 staining in monocyte subsets (*n* = 6). Data were statistically analyzed by ordinary one-way ANOVA with Tukey’s multiple comparison tests **(B)** or Student’s t-test **(E)**, and *p*-values are shown. Data are shown as individual points and as means ± SD for each experimental group **(B, E)**.

The anti-TLR7 mAb abolished splenomegaly in NZBWF1 mice manifesting as spleen weight and splenocyte numbers (**Figures 3A–C**). We analyzed TLR7-dependent cellular changes using flow cytometry. Among the immune cells in the spleen, the anti-TLR7 mAb decreased the frequencies of B cells and monocytes, not those of cDCs, pDCs, granulocytes, NK cells, and T cells (**Figure 3D**). The absolute numbers of B cells, T cells, monocytes and plasma cells were decreased by anti-TLR7 treatment (**Figure 3E**). T cell decrease would be due to impaired activation of B cells and cDCs. Because changes in monocytes were apparent as those in B cells, we focused on monocyte subsets, Ly6C^hi^ classical, and Ly6C^low^ patrolling monocytes (21). These subsets were defined by cell surface markers such as F4/80, CD43, CX3CR1 (**Supplementary Figure 3**). The anti-TLR7 mAb significantly decreased ratios (%) of CD11b^+^ Ly6C^low^ FcγRIV^+^ patrolling monocytes, but did not affect those of Ly6C^hi^ classical monocytes (**Figures 4A–C**) (21, 22).

We explored TLR7 and TLR9 expression of these monocyte subsets in NZBWF1 mice. The expression of TLR7 increased, whereas that of TLR9 decreased with maturation from classical, to patrolling monocytes (21) in NZBWF1 mice (**Figures 4D, E**). Although increased TLR7 expression in patrolling monocytes might be consistent with their pathogenic roles in these mice, such changes in TLR7 and TLR9 expression were also observed in C57BL/6 mice (**Figures 4D, E**).

### Anti-TLR7 mAb Inhibited Disease-Associated Increases in Patrolling Monocytes in the Circulation and Glomeruli

**Figure 5:**
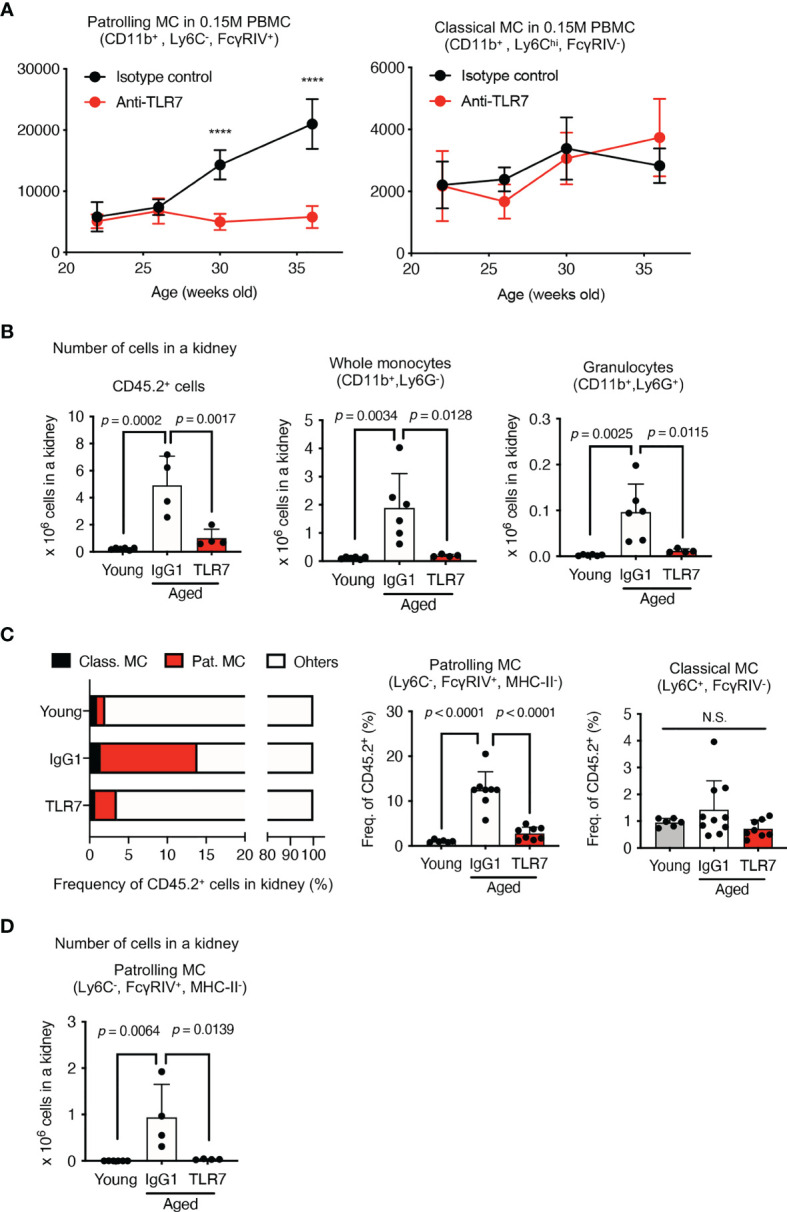
Anti-TLR7 mAb inhibited patrolling monocyte increases in circulation and kidneys. **(A–D)** NZBWF1 mice were administered saline, IgG1 or anti-TLR7 mAb from age 12-16, to 35-40 weeks. **(B–D)** Young (pre-onset) NZBWF1 mice without treatment aged 12 weeks. **(A)** Average numbers of patrolling and classical monocytes in 15 x 10^4^ PBMCs of NZBWF1 mice administered control IgG1 (*n* = 4) or anti-TLR7 mAb (*n* = 7). **(B)** Numbers of CD45.2^+^ leukocytes, CD11b^+^ Ly6G^-^, and CD11b^+^ Ly6G^+^ monocytes in kidneys. Young mice (*n* = 6), NZBWF1 mice treated with control IgG1 (*n* ≥ 4), or anti-TLR7 mAb (*n* ≥ 4). **(C)** Ratios (%) of classical monocytes, patrolling monocytes, and other leukocytes in the kidney. **(D)** Numbers of patrolling monocytes in kidneys. Data were statistically analyzed by 2-way ANOVA **(A)** or ordinary one-way ANOVA **(B–D)**. For 2-way ANOVA, Sidak’s multiple comparisons test was performed to calculate the row factor at each age. ****p < 0.0001; N.S., not significant. For ordinary one-way ANOVA, the results found significant by ANOVA (*p* < 0.05) were further assessed by Tukey’s multiple comparison tests. Data are shown as individual points and as means ± SD for each experimental group.

Classical monocytes mature in bone marrow and enter the circulation (21), where they mature into patrolling monocytes that are regarded as blood macrophages that TLR7-dependently clear damaged endothelial cells (22). We analyzed the numbers of patrolling monocytes in the peripheral blood of NZBWF1 mice that had been administered control IgG1 or the anti-TLR7 mAb. We found TLR7-dependent increases in the number of circulating patrolling monocytes in NZBWF1 mice at the ages of 30 and 37 weeks (**Figure 5A**), much later than the beginning of autoantibody production. Because the anti-TLR7 mAb did not decrease patrolling monocytes, the effect of the anti-TLR7 mAb is unlikely cytotoxic. TLR7 activation was probably required for the increases in these monocytes in mice with lupus, but not in healthy mice, and the anti-TLR7 mAb inhibited TLR7-dependent increase of monocytes. In contrast, the number of classical monocytes in peripheral blood did not increase even at the age of 37 weeks, and the anti-TLR7 mAb did not affect them (**Figure 5A**). These results suggested that TLR7-activation in NZBWF1 patrolling monocytes occurs after their maturation from classical monocytes.

We compared monocytes in the kidneys of young (pre-onset) NZBWF1 mice aged 12 weeks, with those of elderly mice aged 35-40 weeks that were administered IgG1 or anti-TLR7 mAb. The number of immune CD45.2^+^ cells, CD11b^+^ monocytes, and granulocytes were increased in the NZBWF1 mice given IgG1, and anti-TLR7 mAb inhibited this increase (**Figure 5B**). Among the immune cells in the kidney, the ratios (%) and numbers of patrolling monocytes significantly and TLR7-dependently increased (**Figures 5C, D**). These results showed that the numbers of patrolling monocytes TLR7-dependently increased in the kidney and the circulation.

**Figure 6:**
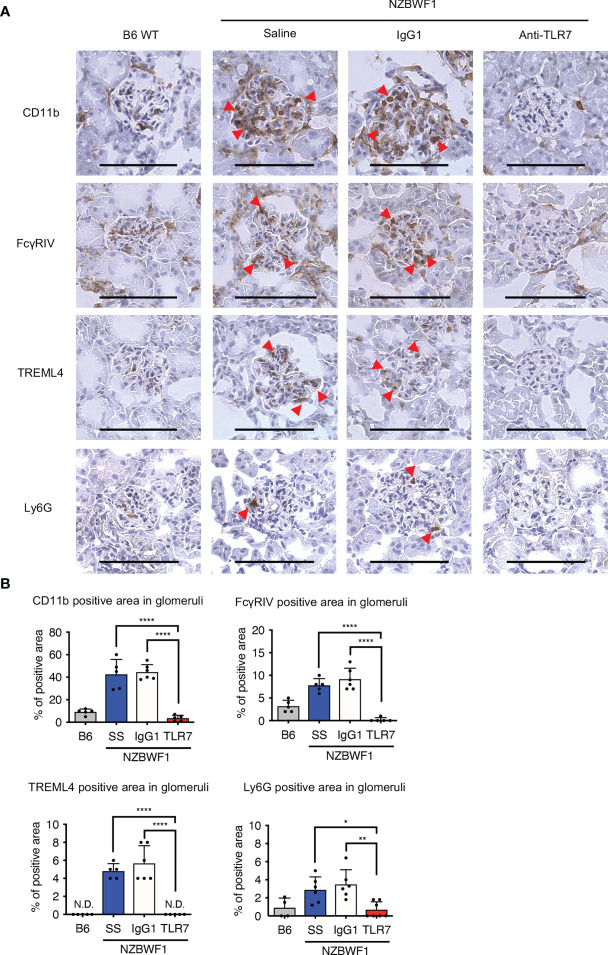
Anti-TLR7 mAb inhibited patrolling monocyte infiltration into glomeruli. **(A, B)** NZBWF1 mice were administered saline, IgG1 or anti-TLR7 mAb from age 12-16, to 35-40 weeks and compared with age-matched B6 mice. **(A)** Representative images show immunohistological findings of glomerular myeloid cells expressing CD11b, FcγRIV, TREML4 or Ly6G. Red arrowheads, positively stained cells. Scale bar, 100 μm. **(B)** Ratios of stained areas in each glomerulus statistically analyzed using fluorescence microscopy. Numbers of glomeruli assessed (5-10 from one kidney in B6 (*n* ≥ 4), saline (*n* = 5), control IgG1 (*n* = 6), and anti-TLR7 mAb (*n* ≥ 5) **(A, B)**. Data were statistically analyzed using one-way ANOVA with Tukey’s multiple comparison tests. *p < 0.05, **p < 0.01, ****p < 0.0001. N.D., not detected. Data are shown as individual points and as means ± SD for each experimental group.

We used immunohistochemical staining to determine whether abundant patrolling monocytes infiltrated the glomeruli. The ratio (%) of glomeruli containing CD11b^+^ cells in elderly NZBWF1 mice was ~ 40%, compared with those in age-matched C57BL/6 mice, which were < 10% (**Figures 6A, B**). The anti-TLR7 mAb decreased the number of glomerular CD11b^+^ cells (**Figures 6A, B**). To confirm that patrolling monocytes infiltrated the glomeruli, we stained glomeruli for FcγRIV and TREML4, which are expressed at high levels in patrolling monocytes (23, 24). We also stained Ly6G to detect infiltrative neutrophils in glomeruli, because neutrophils were increased in the kidneys of NZBWF1 mice (**Figure 5B**). The expression of FcγRIV and TREML4 suggested that patrolling monocytes infiltrated the glomeruli of NZBWF1 mice (**Figures 6A, B**). Although Ly6G^+^ neutrophils were detected in significant numbers of glomeruli, the ratios (%) of Ly6G^+^ glomeruli were much lower than those of patrolling monocytes (**Figure 6B**). The anti-TLR7 mAb significantly decreased neutrophil recruitment to the glomeruli. These results might be consistent with the finding that patrolling monocytes recruit neutrophils to cause endothelial damage (22). Neutrophil-independent damage might occur in glomeruli with patrolling monocytes but without neutrophils. These results suggested that anti-TLR7 mAb mitigates lupus nephritis by inhibiting the accumulation of patrolling monocytes in glomeruli.

### Expression of Lupus-Associated Genes in Patrolling Monocytes in NZBWF1 Mice

**Figure 7:**
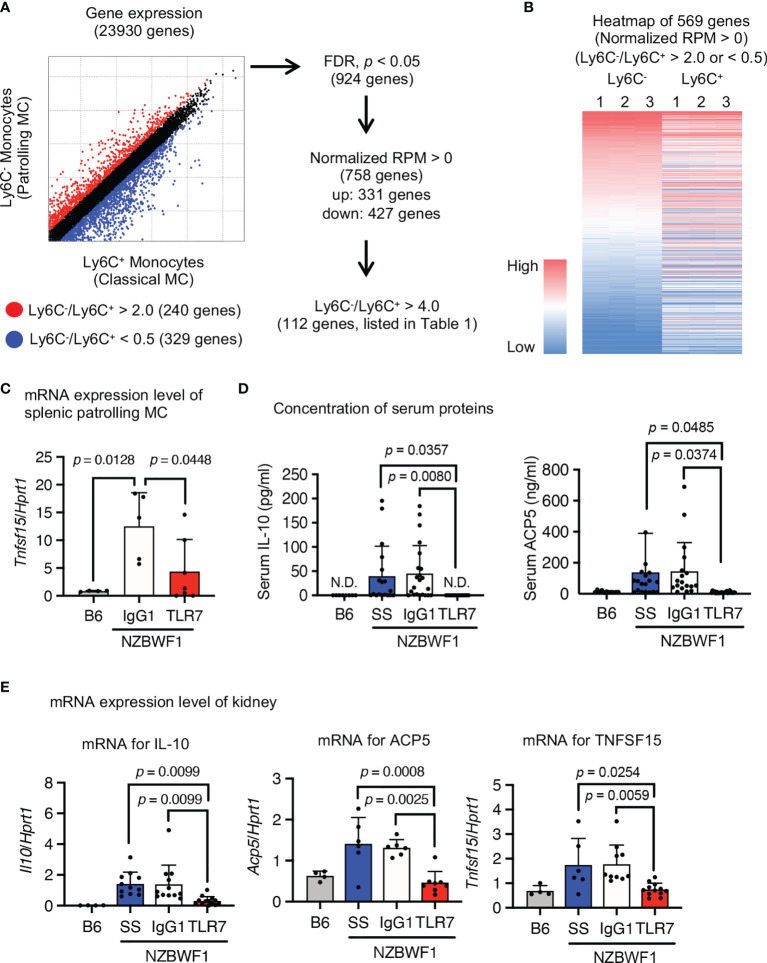
Expression of SLE-associated genes in patrolling monocytes in NZBWF1 mice. Expression of 23,930 analyzed genes in patrolling monocytes and classical monocytes of NZBWF1 mice aged 30-35 weeks. Red and blue dots show genes with expression ratios in patrolling monocytes compared with classical monocytes that were > 2 or < 0.5, respectively. We identified 924 significantly changed genes (*p* < 0.05; Benjamin-Hochberg FDR tests), and 758 with normalized expression (reads per million, RPM) > 0. Among them, the expression of 331 genes was considered as upregulated in patrolling monocytes compared with classical monocytes. Among these 331 genes, 112 with a patrolling/classical expression ratio > 4 are shown in **Supplementary Table 1**. **(B)** Heatmap of RPM of 569 genes (red and blue dots in **(A)** in descending order based on that of patrolling monocytes. Results are from individual samples (1–3). **(C–E)** NZBWF1 mice administered saline, IgG1 or anti-TLR7 mAb from age 12-16 to 35-40 weeks compared with age-matched B6 mice. **(C, E)** Expression of mRNAs encoding indicated genes in splenic patrolling monocytes **(C)** and kidney **(E)** were analyzed by real-time PCR. Results are normalized by *Hprt1* mRNA. **(D)** Concentration of IL-10 and ACP5 in serum measured by ELISA. B6 (*n* = 4), saline (*n* ≥ 6), control IgG1 (*n* ≥ 5), and anti-TLR7 mAb (*n* ≥ 6). Data were statistically analyzed using one-way ANOVA. The results found significant by ANOVA (*p* < 0.05) were further assessed by Tukey’s multiple comparison tests and the *p*-values are shown **(C–E)**. Data are shown as individual points and as means ± SD for each experimental group **(C–E)**.

We characterized the abundant patrolling monocytes in NZBWF1 mice using transcriptome analysis. We compared Ly6C^hi^ classical and Ly6C^low^ patrolling monocytes because classical monocytes were not increased in NZBWF1 mice (**Figures 4B**, **5A**). The expression of 924 genes significantly differed between patrolling monocytes and classical monocytes. Among them, 112 genes were upregulated > 4-fold in patrolling monocytes (**Figures 7A, B**, and **Supplementary Table 1**). Some of these upregulated genes (*Fabp4, Il10, Pecam1*, *Pparg*, *Vwf*, *Cd36*, *Cd274*, and *Tnfsf15*), are upregulated in SLE as well (25–30). Although loss-of-function mutations of *Acp5* cause spondyloenchon-drodysplasia with immune dysregulation (SPENCDI), which is a skeletal and neurological disorder with lupus-like symptoms and a type I interferon signature (31), the expression of *Acp5* mRNA increased in patrolling monocytes in NZBWF1 mice.

To determine whether these SLE-associated genes are TLR7-dependently upregulated, we measured levels of mRNAs encoding SLE-associated genes in isolated patrolling monocytes using real-time PCR. The anti-TLR7 mAb decreased *Tnfsf15* expression in splenic patrolling monocytes (**Figure 7C**). Although the anti-TLR7 mAb did not significantly change levels of mRNAs encoding the other genes, it returned the elevated serum IL-10 and ACP5 to normal levels (**Figure 7D**). Anti-TLR7 mAb might impact monocytes during transcription and post-transcriptional processes. We examined the expression of mRNAs encoding these upregulated genes in the kidney using real-time PCR. The anti-TLR7 mAb decreased the upregulated mRNAs encoding *Il10, Acp5*, and *Tnfsf15* in NZBWF1 mice to the levels in C57BL/6 kidneys (**Figure 7E**). Expression of mRNAs encoding inflammatory cytokines and type I interferons were not changed by the anti-TLR7 mAb (**Supplementary Figure 4**). These results suggested that patrolling monocytes TLR7-dependently expressing lupus-associated genes such as *Il10, Acp5*, and *Tnfsf15* infiltrated the kidneys of NZBWF1 mice.

### Expression of TLR7-Dependent Cell Surface Markers on Patrolling Monocytes

**Figure 8:**
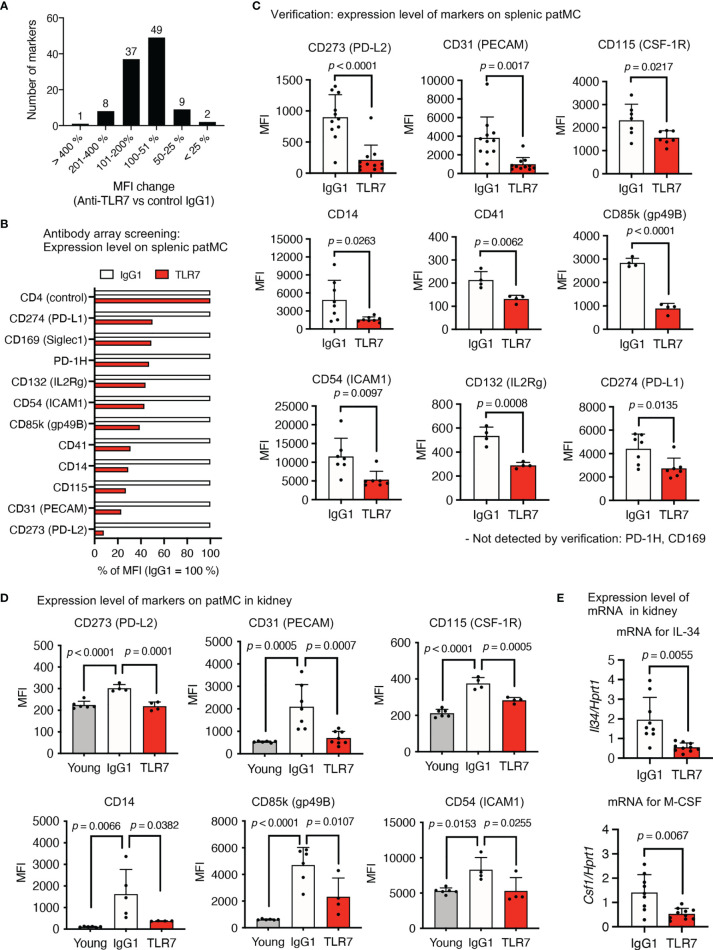
TLR7-dependent expression of the cell surface molecules on patrolling monocytes in NZBWF1 mice. **(A–E)** NZBWF1 mice were administered saline, IgG1 or the anti-TLR7 mAb from age of 12-16 to 35-40 weeks. Young (pre-onset) untreated NZBWF1 mice were analyzed at 12 weeks of age. **(A)** Splenic patrolling monocytes were stained using LEGENDScreen antibody array. Distribution of ratios (%) of anti-TLR7 to IgG1 treatment is shown. **(B)** Mean fluorescent intensity of markers with > 50% less expression caused by anti-TLR7. Results are average values from four mice. **(C)** Expression of markers on patrolling monocytes in spleen. Control IgG1 (*n* ≥ 4), and anti-TLR7 mAb (*n* ≥ 4). **(D)** Expression of markers on patrolling monocytes in kidneys. Young (pre-onset) 12-week-old NZBWF1 mice were controls. Young mice (*n* = 6), control IgG1 (*n* ≥ 4), and anti-TLR7 (*n* ≥ 4). **(E)** Expression of mRNA encoding IL-34 and M-CSF in kidneys analyzed by real-time PCR. Results are normalized to *Hprt1* mRNA. Control IgG1 (*n* ≥ 7), and anti-TLR7 (*n* ≥ 6). Data were statistically analyzed by Student’s t-tests, Welch’s t-tests **(C, E)**, or one-way ANOVA **(D)**. The results found significant by ANOVA (*p* < 0.05) were further assessed by Tukey’s multiple comparison tests. Data are shown as individual points and as means ± SD for each experimental group **(C–E)**.

We applied an antibody array system to identify the TLR7-dependent expression of a cell surface marker in patrolling monocytes, because the anti-TLR7 mAb might act on monocytes at the post-transcriptional level. Flow cytometry using 258 antibodies revealed that 106 markers were expressed on patrolling monocytes. The mean fluorescence intensity (MFI) of 60 markers was reduced by the anti-TLR7 mAb, and we focused on 11 among them that were reduced > 50% by the anti-TLR7 mAb (**Figures 8A, B**). Splenic patrolling monocytes were stained with these antibodies for verification, and the MFIs of the markers CD273 (PD-L2), CD31 (platelet endothelial cell adhesion molecule 1; PECAM), CD115 (CSF1-R), CD14, CD41, CD85k (GP49B), CD54 (ICAM1), CD132, and CD274 (PD-L1) were significantly decreased by the anti-TLR7 mAb (**Figure 8C**).

We compared patrolling monocytes in the kidneys of young (pre-onset) and elderly NZBWF1 mice administered IgG1 or the anti-TLR7 mAb, to determine the expression of these markers in patrolling monocytes in the kidney. The expression of CD273, CD31 (PECAM), CD115, CD14, CD85k, and CD54 (ICAM1) was significantly increased by disease progression and was sensitive to the anti-TLR7 mAb (**Figure 8D**). These markers might play pathogenic roles in lupus nephritis. For example, the CD115 inhibitor GW2580 attenuates nephritis and neuropsychiatric diseases in the lupus-prone mouse strain, MRL-lpr/lpr (31). In this context, the fact that mRNAs encoding M-CSF and IL-34, ligands for CD115, were TLR7-dependently increased in the kidneys of NZBWF1 mice is notable (**Figure 8E**). As these cytokines are produced by mesangial cells and podocytes in lupus nephritis (32), glomerular infiltration by CD115^+^ patrolling monocytes might upregulate the expression of mRNAs encoding CD115 ligands, leading to a vicious circle between patrolling monocytes and glomerulus-intrinsic cells such as mesangial cells and podocytes.

## Discussion

Our results showed that TLR7 activation increased the number of patrolling monocytes in the spleen, circulation, and kidneys of NZBWF1 mice. Lupus-associated TLR7-dependent monocytosis has been identified in the *Yaa* mouse model of lupus (33), in which TLR7 is hyperactivated due to its gene duplication (10, 11). Furthermore, the numbers of patrolling monocytes in the kidney are increased in lupus-prone MRL/*lpr*, B6.*Sle yaa*, and *Tnip1* ^-/-^ mice (6). Patrolling monocytes infiltrate the glomeruli of patients with SLE (6, 22, 34). These findings suggest that increased numbers of patrolling monocytes directly drive lupus nephritis. Consistent with this, lupus nephritis in B6.*Sleyaa* and *Tnip1* ^-/-^ mice is ameliorated by preventing monocyte maturation into patrolling monocytes by deleting their master transcription factor NR4A1 (6, 35). Our anti-TLR7 mAb findings suggested that TLR7 drives lupus-associated increases in patrolling monocytes in NZBWF1 mice. Because the anti-TLR7 mAb did not impair homeostatic monocyte maturation into patrolling monocytes, TLR7 was activated only in a subpopulation of patrolling monocytes that infiltrated the kidney of NZBWF1 mice. Macrophage-specific *Tnip1* deletion is sufficient for nephritis to develop in *Tnip1* ^-/-^ mice, and high levels of TLR7 were expressed in patrolling monocytes of NZBWF1 mice. These results suggest that patrolling monocytes are increased by cell-autonomous TLR7 activation in these mice. Endogenous retroelements, such as Alu RNAs, might activate TLR7 in patrolling monocytes (36). A mechanism through which TLR7 promotes monocytosis might be suggested by our finding of increased CD115 expression in patrolling monocytes. Because CD115 delivers a survival signal in patrolling monocytes (37), upregulated CD115 activation might underlie lupus-associated monocytosis in NZBWF1 mice.

Lupus-associated increases in patrolling monocytes were detected not only in the circulation, where classical monocytes mature into patrolling monocytes (21), but also in the spleen and kidney. Circulating patrolling monocytes are likely to infiltrate these organs. Transcriptome analyses and an antibody array showed a TLR7-dependent increase in CD31, which belongs to the immunoglobulin superfamily and is expressed at the intercellular junctions of endothelial cells and leukocytes including monocytes (38). Homophilic interactions between leukocyte CD31 and endothelial CD31 enable trans-endothelial leukocyte migration (39). Thus, patrolling monocytes might use CD31 to migrate into the glomeruli of NZBWF1 mice. The expression of CD31 in the peripheral blood of patients positively correlates with SLE disease activity (29); therefore, CD31 expression might also increase TLR7-dependently in human SLE.

Messenger RNAs encoding M-CSF and IL-34 that are ligands for CD115, were TLR7-dependently increased in the kidneys of NZBWF1 mice. These ligands are produced by mesangial cells and podocytes, respectively, in glomeruli (32), indicating that glomerular patrolling monocytes could change the microenvironment to drive glomerulonephritis. Consistent with this, the CD115 inhibitor GW2580 attenuates nephritis and neuropsychiatric disease in a lupus-prone mouse strain MRL-lpr/lpr (40). The molecular mechanisms through which patrolling monocytes drive glomerular damage, might be *via* neutrophil recruitment, which results in endothelial cell damage (22). We detected TLR7-dependent increases in neutrophils in the kidneys, but considerably fewer neutrophils than patrolling monocytes infiltrated the glomeruli of NZBWF1 mice. Patrolling monocytes are likely to drive nephritis without neutrophil recruitment when glomeruli do not harbor neutrophils. Notably, patrolling monocytes TLR7-dependently produced IL-10. Although large amounts of IL-10 are produced by various types of immune cells such as CD4^+^ T cells and DCs in NZBWF1 mice (41), the anti-TLR7 mAb significantly decreased serum IL-10 and IL-10 mRNA in the kidneys, suggesting that patrolling monocytes are a major source of IL-10 in NZBWF1 mice. Interleukin-10 is a pleiotropic cytokine that mediates both immunostimulatory and immunoregulatory effects in humans and mice (42). The anti-IL-10 mAb delays disease development in NZBWF1 mice, whereas IL-10 accelerates disease progression (17). Elevated levels of serum IL-10 have also been found in other kidney diseases, such as mesangioproliferative glomerulonephritis, IgA nephropathy, and diabetic nephropathy (40). As IL-10 promotes glomerular damage by increasing mesangial proliferation and the mesangial deposition of immune complexes (43, 44), our results suggested that the TLR7-dependent production of IL-10 in glomerular patrolling monocytes promotes lupus nephritis. Tumor necrosis factor superfamily 15 (TNFSF15), also known as TNF-like ligand 1A (TL1A) or vascular endothelial growth inhibitor (VEGI), is produced by monocytes and macrophages stimulated with immune complexes (ICs) but not by TLR ligands, including the TLR7/8 ligand R848 (45), which inhibits IC-dependent TNFSF15 expression in human peripheral blood monocytes (46). The activation of TLR7 in NZBWF1 mice might be distinct from R848-mediated TLR7/8 activation in human peripheral blood monocytes. The Y RNA binding protein, Ro60, is an autoantigen that binds to endogenous retroelements such as Alu RNAs, which might activate TLR7 (36). Immune complexes containing Alu RNA-Ro60 might act on cell surface FcRs and subsequently on endosomal TLR7 in patrolling monocytes to induce TNFSF15, which acts on death receptor-3 expressed on endothelial cells in kidneys (47). Therefore, TNFSF15 might contribute to glomerular damage in NZBWF1 mice.

Because deleting TLR7 is not sufficient to rescue MRL/*lpr* or *Tnip1* ^-/-^ mice from nephritis (6, 7), other TLRs such as TLR9 might also drive the increases in lupus-associated patrolling monocytes in these mice (6). In this context, TLR7-dependent increase in serum ACP5, also known as tartrate-resistant acid phosphatase (TRAP) is notable. High levels of ACP5 are expressed in osteoclasts, and serum ACP5 is considered a marker of osteoclast activation. Our results suggested that serum ACP5 could be a marker of TLR7-dependent monocyte activation in SLE. On the other hand, biallelic loss-of-function mutations cause spondyloenchondrodysplasia with immune dysregulation (SPENCDI), a skeletal and neurological disorder with lupus-like symptoms and a type I interferon signature (31), in which TLR9 hyperactivation is suggested to cause immune dysregulation. If SLE can be subdivided into one type that depends on TLR7 and another that depends on TLR9, they might be distinguished from each other by serum ACP5.

The CD115 inhibitor GW2580 inhibits monocyte accumulation in the kidneys, but not IgG deposition in glomeruli of MRL/*lpr* mice (40). Lack of the FcRγ chain impairs the activation of FcR^+^ monocytes, thus protecting NZBWF1 mice from lupus nephritis, despite unaltered immune complex deposition in glomeruli (4, 5). These previous studies suggest that monocytes/macrophages are activated independently of B cell activation. Because autoreactive B cell also plays pathogenic roles in NZBWF1 mice, B cell is a therapeutic target for the control of lupus nephritis. The anti-TLR7 mAb inhibited both monocytosis and IgG deposition, suggesting that the anti-TLR7 mAb acted on both monocytes and B cells. The anti-TLR7 mAb also inhibits TLR7 responses in BM-cDCs and BM-pDCs *in vitro* (16), which might explain the decrease in number of splenic T cells by the anti-TLR7 mAb. We concluded that TLR7 is a promising therapeutic target for controlling SLE, and a mAb against TLR7 is a promising modality with which various immune cells can be targeted.

## Data Availability Statement

The original contributions presented in the study are publicly available. This data can be found here: National Center for Biotechnology Information (NCBI) BioProject database under accession number PRJDB12445 and in DDBJ under accession number DRA012916.

## Ethics Statement

The animal study was reviewed and approved by Animal Experiment Committee of The Institute of Medical Science, The University of Tokyo.

## Author Contributions

YMu, RF, and KM conceived and designed the experiments. YMu, RF, and KM wrote the manuscript. YMu, RF, RT, YMo, AK, RS, and HA performed the experiments. YMu, RF, YMo, and AK generated the antibodies. KY and YF conducted and performed RNA sequencing analysis. HA, NT, HS, YS, and NY analyzed the data with clinical insights. All the authors reviewed and edited the results and comments on the manuscript. YMu and RF contributed equally as co-first authors. All authors contributed to the article and approved the submitted version.

## Funding

This work was supported in part by Grant-in-Aid for Scientific Research (S) and (A) to KM (16H06388, 21H04800); Grant-in-Aid for Scientific Research (C) to RF (18K07169); Grant-in-Aid for Scientific Research on Innovative Areas to KM (18H04666); Grant-in-Aid for Early-Career Scientists to YMu (20K16227); AMED-CiCLE; Joint Research Project of the Institute of Medical Science at the University of Tokyo.

## Conflict of Interest

The authors declare that the research was conducted in the absence of any commercial or financial relationships that could be construed as a potential conflict of interest.

## Publisher’s Note

All claims expressed in this article are solely those of the authors and do not necessarily represent those of their affiliated organizations, or those of the publisher, the editors and the reviewers. Any product that may be evaluated in this article, or claim that may be made by its manufacturer, is not guaranteed or endorsed by the publisher.

## References

[1] Yu F Haas M Glassock R Zhao MH. Redefining Lupus Nephritis: Clinical Implications of Pathophysiologic Subtypes. Nat Rev Nephrol (2017) 13(8):483–95. doi: 10.1038/nrneph.2017.85 28669995

[2] Smith CK Kaplan MJ. The Role of Neutrophils in the Pathogenesis of Systemic Lupus Erythematosus. Curr Opin Rheumatol (2015) 27(5):448–53. doi: 10.1097/BOR.0000000000000197 PMC12351535 26125102

[3] Tsokos GC Lo MS Reis PC Sullivan KE. New Insights Into the Immunopathogenesis of Systemic Lupus Erythematosus. Nat Rev Rheumatol (2016) 12(12):716–30. doi: 10.1038/nrrheum.2016.186 27872476

[4] Clynes R Dumitru C Ravetch JV. Uncoupling of Immune Complex Formation and Kidney Damage in Autoimmune Glomerulonephritis. Science (1998) 279(5353):1052–4. doi: 10.1126/science.279.5353.1052 9461440

[5] Bergtold A Gavhane A D’Agati V Madaio M Clynes R. FcR-Bearing Myeloid Cells Are Responsible for Triggering Murine Lupus Nephritis. J Immunol (2006) 177(10):7287–95. doi: 10.4049/jimmunol.177.10.7287 17082647

[6] Kuriakose J Redecke V Guy C Zhou J Wu R Ippagunta SK. Patrolling Monocytes Promote the Pathogenesis of Early Lupus-Like Glomerulonephritis. J Clin Invest (2019) 129(6):2251–65. doi: 10.1172/JCI125116 PMC6546471 31033479

[7] Christensen SR Shupe J Nickerson K Kashgarian M Flavell RA Shlomchik MJ. Toll-Like Receptor 7 and TLR9 Dictate Autoantibody Specificity and Have Opposing Inflammatory and Regulatory Roles in a Murine Model of Lupus. Immunity (2006) 25(3):417–28. doi: 10.1016/j.immuni.2006.07.013 16973389

[8] Santiago-Raber ML Dunand-Sauthier I Wu T Li QZ Uematsu S Akira S. Critical Role of TLR7 in the Acceleration of Systemic Lupus Erythematosus in TLR9-Deficient Mice. J Autoimmun (2010) 34(4):339–48. doi: 10.1016/j.jaut.2009.11.001 19944565

[9] Jiang W Zhu F-G Bhagat L Yu D Tang JX Kandimalla ER. A Toll-Like Receptor 7, 8, and 9 Antagonist Inhibits Th1 and Th17 Responses and Inflammasome Activation in a Model of IL-23-Induced Psoriasis. J Invest Dermatol (2013) 133(7):1777–84. doi: 10.1038/jid.2013.57 23370539

[10] Pisitkun P Deane JA Difilippantonio MJ Tarasenko T Satterthwaite AB Bolland S. Autoreactive B Cell Responses to RNA-Related Antigens Due to TLR7 Gene Duplication. Science (2006) 312(5780):1669. doi: 10.1126/science.1124978 16709748

[11] Subramanian S Tus K Li Q-Z Wang A Tian X-H Zhou J. A Tlr7 Translocation Accelerates Systemic Autoimmunity in Murine Lupus. Proc Natl Acad Sci USA (2006) 103(26):9970–5. doi: 10.1073/pnas.0603912103 PMC1502563 16777955

[12] Goel RR Wang X O’Neil LJ Nakabo S Hasneen K Gupta S. Interferon Lambda Promotes Immune Dysregulation and Tissue Inflammation in TLR7-Induced Lupus. Proc Natl Acad Sci USA (2020) 117(10):5409–19. doi: 10.1073/pnas.1916897117 PMC7071891 32094169

[13] Yokogawa M Takaishi M Nakajima K Kamijima R Fujimoto C Kataoka S. Epicutaneous Application of Toll-Like Receptor 7 Agonists Leads to Systemic Autoimmunity in Wild-Type Mice: A New Model of Systemic Lupus Erythematosus. Arthritis Rheumatol (2014) 66(3):694–706. doi: 10.1002/art.38298 24574230

[14] Tojo S Zhang Z Matsui H Tahara M Ikeguchi M Kochi M. Structural Analysis Reveals TLR7 Dynamics Underlying Antagonism. Nat Commun (2020) 11(1):5204. doi: 10.1038/s41467-020-19025-z 33060576 PMC7562955

[15] Sato R Reuter T Hiranuma R Shibata T Fukui R Motoi Y. The Impact of Cell Maturation and Tissue Microenvironments on the Expression of Endosomal Toll-Like Receptors in Monocytes and Macrophages. Int Immunol (2020) 32(12):785–98. doi: 10.1093/intimm/dxaa055 32840578

[16] Kanno A Tanimura N Ishizaki M Ohko K Motoi Y Onji M. Miyake: Targeting Cell Surface TLR7 for Therapeutic Intervention in Autoimmune Diseases. Nat Commun (2015) 6:6119. doi: 10.1038/ncomms7119 25648980

[17] Ishida H Muchamuel T Sakaguchi S Andrade S Menon S Howard M. Continuous Administration of Anti-Interleukin 10 Antibodies Delays Onset of Autoimmunity in NZB/W F1 Mice. J Exp Med (1994) 179(1):305–10. doi: 10.1084/jem.179.1.305 PMC2191319 8270873

[18] Murakami Y Fukui R Motoi Y Shibata T Saitoh S-I Sato R. The Protective Effect of the Anti-Toll-Like Receptor 9 Antibody Against Acute Cytokine Storm Caused by Immunostimulatory DNA. Sci Rep (2017) 7:44042. doi: 10.1038/srep44042 28266597 PMC5339793

[19] Hwang S-H Lee H Yamamoto M Jones LA Dayalan J Hopkins R. B Cell TLR7 Expression Drives Anti-RNA Autoantibody Production and Exacerbates Disease in Systemic Lupus Erythematosus–Prone Mice. J Immunol (2012) 189(12):5786. doi: 10.4049/jimmunol.1202195 23150717 PMC3544945

[20] Shibata T Ohto U Nomura S Kibata K Motoi Y Zhang Y. Guanosine and Its Modified Derivatives Are Endogenous Ligands for TLR7. Int Immunol (2016) 28(5):211–22. doi: 10.1093/intimm/dxv062 PMC4888345 26489884

[21] Ginhoux F Jung S. Monocytes and Macrophages: Developmental Pathways and Tissue Homeostasis. Nat Rev Immunol (2014) 14(6):392–404. doi: 10.1038/nri3671 24854589

[22] Carlin LM Stamatiades EG Auffray C Hanna RN Glover L Vizcay-Barrena G. Nr4a1-Dependent Ly6Clow Monocytes Monitor Endothelial Cells and Orchestrate Their Disposal. Cell (2013) 153(2):362–75. doi: 10.1016/j.cell.2013.03.010 PMC3898614 23582326

[23] Briseño CG Haldar M Kretzer NM Wu X Theisen DJ Kc W. Distinct Transcriptional Programs Control Cross-Priming in Classical and Monocyte-Derived Dendritic Cells. Cell Rep (2016) 15(11):2462–74. doi: 10.1016/j.celrep.2016.05.025 PMC4941620 27264183

[24] Biburger M Aschermann S Schwab I Lux A Albert H Danzer H. Monocyte Subsets Responsible for Immunoglobulin G-Dependent Effector Functions *In Vivo* . Immunity (2011) 35(6):932–44. doi: 10.1016/j.immuni.2011.11.009 22169040

[25] Godsell J Rudloff I Kandane-Rathnayake R Hoi A Nold MF Morand EF. Clinical Associations of IL-10 and IL-37 in Systemic Lupus Erythematosus. Sci Rep (2016) 6:34604. doi: 10.1038/srep34604 27708376 PMC5052569

[26] Parra S Cabré A Marimon F Ferré R Ribalta J Gonzàlez M. Circulating FABP4 Is a Marker of Metabolic and Cardiovascular Risk in SLE Patients. Lupus (2014) 23(3):245–54. doi: 10.1177/0961203313517405 24390652

[27] Xu WD Chen DJ Li R Ren CX Ye DQ. Elevated Plasma Levels of TL1A in Newly Diagnosed Systemic Lupus Erythematosus Patients. Rheumatol Int (2015) 35(8):1435–7. doi: 10.1007/s00296-015-3277-2 25929716

[28] Oxer DS Godoy LC Borba E Lima-Salgado T Passos LA Laurindo I. Pparγ Expression Is Increased in Systemic Lupus Erythematosus Patients and Represses CD40/CD40L Signaling Pathway. Lupus (2011) 20(6):575–87. doi: 10.1177/0961203310392419 21415255

[29] da Rosa Franchi Santos LF Stadtlober NP Costa Dall’Aqua LG Scavuzzi BM Guimarães PM Flauzino T. Increased Adhesion Molecule Levels in Systemic Lupus Erythematosus: Relationships With Severity of Illness, Autoimmunity, Metabolic Syndrome and Cortisol Levels. Lupus (2018) 27(3):380–8. doi: 10.1177/0961203317723716 29400123

[30] Lee MH Gallo PM Hooper KM Corradetti C Ganea D Caricchio R. The Cytokine Network Type I IFN-IL-27-IL-10 Is Augmented in Murine and Human Lupus. J Leukoc Biol (2019) 106(4):967–75. doi: 10.1002/JLB.3AB0518-180RR PMC7901002 31216373

[31] Briggs TA Rice GI Daly S Urquhart J Gornall H Bader-Meunier B. Tartrate-Resistant Acid Phosphatase Deficiency Causes a Bone Dysplasia With Autoimmunity and a Type I Interferon Expression Signature. Nat Genet (2011) 43(2):127–31. doi: 10.1038/ng.748 PMC3030921 21217755

[32] Sung SJ Fu SM. Interactions Among Glomerulus Infiltrating Macrophages and Intrinsic Cells *via* Cytokines in Chronic Lupus Glomerulonephritis. J Autoimmun (2020) 106:102331. doi: 10.1016/j.jaut.2019.102331 31495649 PMC6930355

[33] Amano H Amano E Santiago-Raber ML Moll T Martinez-Soria E Fossati-Jimack L. Selective Expansion of a Monocyte Subset Expressing the CD11c Dendritic Cell Marker in the Yaa Model of Systemic Lupus Erythematosus. Arthritis Rheum (2005) 52(9):2790–8. doi: 10.1002/art.21365 16142734

[34] Cros J Cagnard N Woollard K Patey N Zhang SY Senechal B. Human CD14dim Monocytes Patrol and Sense Nucleic Acids and Viruses *via* TLR7 and TLR8 Receptors. Immunity (2010) 33(3):375–86. doi: 10.1016/j.immuni.2010.08.012 PMC3063338 20832340

[35] Hanna RN Carlin LM Hubbeling HG Nackiewicz D Green AM Punt JA. The Transcription Factor NR4A1 (Nur77) Controls Bone Marrow Differentiation and the Survival of Ly6C- Monocytes. Nat Immunol (2011) 12(8):778–85. doi: 10.1038/ni.2063 PMC3324395 21725321

[36] Hung T Pratt GA Sundararaman B Townsend MJ Chaivorapol C Bhangale T. The Ro60 Autoantigen Binds Endogenous Retroelements and Regulates Inflammatory Gene Expression. Science (2015) 350(6259):455. doi: 10.1126/science.aac7442 26382853 PMC4691329

[37] Yona S Kim K-W Wolf Y Mildner A Varol D Breker M. Jung: Fate Mapping Reveals Origins and Dynamics of Monocytes and Tissue Macrophages Under Homeostasis. Immunity (2013) 38(1):79–91. doi: 10.1016/j.immuni.2012.12.001 23273845 PMC3908543

[38] Muller WA. Mechanisms of Leukocyte Transendothelial Migration. Annu Rev Pathol (2011) 6:323–44. doi: 10.1146/annurev-pathol-011110-130224 PMC3628537 21073340

[39] Muller WA Weigl SA Deng X Phillips DM. PECAM-1 Is Required for Transendothelial Migration of Leukocytes. J Exp Med (1993) 178(2):449–60. doi: 10.1084/jem.178.2.449 PMC2191108 8340753

[40] Chalmers SA Wen J Shum J Doerner J Herlitz L Putterman C. CSF-1R Inhibition Attenuates Renal and Neuropsychiatric Disease in Murine Lupus. Clin Immunol (2017) 185:100–8. doi: 10.1016/j.clim.2016.08.019 PMC5326697 27570219

[41] Amend A Wickli N Schäfer AL Sprenger DTL Manz RA Voll RE. Dual Role of Interleukin-10 in Murine NZB/W F1 Lupus. Int J Mol Sci (2021) 22(3):1347–69. doi: 10.3390/ijms22031347 PMC7866297 33572870

[42] Saxena A Khosraviani S Noel S Mohan D Donner T Hamad AR. Interleukin-10 Paradox: A Potent Immunoregulatory Cytokine That has Been Difficult to Harness for Immunotherapy. Cytokine (2015) 74(1):27–34. doi: 10.1016/j.cyto.2014.10.031 25481648 PMC4454631

[43] Chadban SJ Tesch GH Foti R Atkins RC Nikolic-Paterson DJ. Interleukin-10 Is a Mesangial Cell Growth Factor *In Vitro* and *In Vivo* . Lab Invest (1997) 76(5):619–27. 9166281

[44] Sinuani I Beberashvili I Averbukh Z Sandbank J. Role of IL-10 in the Progression of Kidney Disease. World J Transplant (2013) 3(4):91–8. doi: 10.5500/wjt.v3.i4.91 PMC3879528 24392313

[45] Prehn JL Thomas LS Landers CJ Yu QT Michelsen KS Targan SR. The T Cell Costimulator TL1A Is Induced by FcgammaR Signaling in Human Monocytes and Dendritic Cells. J Immunol (2007) 178(7):4033–8. doi: 10.4049/jimmunol.178.7.4033 17371957

[46] Saruta M Michelsen KS Thomas LS Yu QT Landers CJ Targan SR. TLR8-Mediated Activation of Human Monocytes Inhibits TL1A Expression. Eur J Immunol (2009) 39(8):2195–202. doi: 10.1002/eji.200939216 PMC2839407 19637197

[47] Wang J Al-Lamki RS Zhu X Liu H Pober JS Bradley JR. TL1-A Can Engage Death Receptor-3 and Activate NF-Kappa B in Endothelial Cells. BMC Nephrol (2014) 15:178. doi: 10.1186/1471-2369-15-178 25399326 PMC4239315

