# Supplementary material for: Anti-TLR7 Antibody Protects Against Lupus Nephritis in NZBWF1 Mice by Targeting B Cells and Patrolling Monocytes
Source: Front Immunol. 2021 Nov 11;12:777197. doi: 10.3389/fimmu.2021.777197 (PMC8632649; doi:10.3389/fimmu.2021.777197)
Supplement: Supplementary file 1 [file DataSheet_1.pdf]

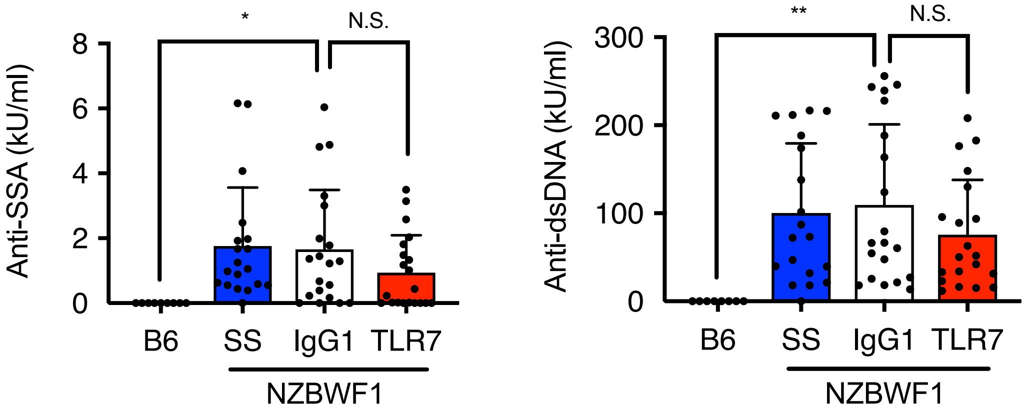

Supplementary Figure 1. Autoantibody production is started at early age of NZBWF1 mice
NZBWF1 mice were administered saline, IgG1 or anti-TLR7 mAb from ages of 12-16 to 20 weeks and compared with age-matched WT C57BL/6 (B6) mice. Serum levels of autoantibodies in mice aged 20 weeks were measured by ELISA. B6 (n = 9), saline (SS, n = 19), control IgG1 (IgG1, n = 20), and anti-TLR7 mAb (TLR7, n = 20).

Data were statistically analyzed using one-way ANOVA with Tukey's multiple comparison tests. * $p < 0.05$, ** $p < 0.01$, N.S., not significant. Data are shown as individual points and as means ± SD for each experimental group.

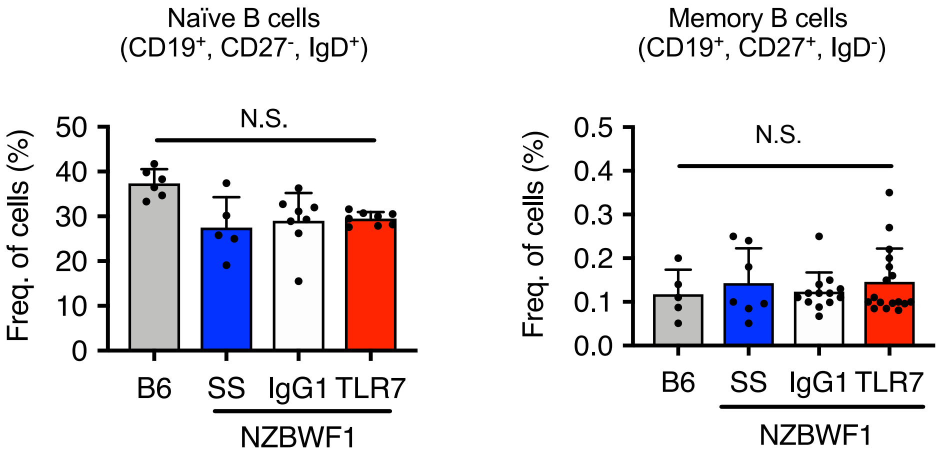

Supplementary Figure 2. Frequency of B cell subset in spleen

NZBWF1 mice were administered saline, IgG1 or anti-TLR7 mAb from ages of 12-16 to 35-40 weeks and compared with age-matched WT C57BL/6 (B6) mice. Ratios of B cell subsets were analyzed by flow cytometry. B6 ($n \geq 5$), Standard saline (SS, $n \geq 5$), control IgG1 (IgG1, $n \geq 8$), and anti-TLR7 mAb (TLR7, $n \geq 8$)

Data were statistically analyzed using one-way ANOVA with Tukey's multiple comparison tests. N.S., not significant. Data are shown as individual points and as means ± SD for each experimental group.

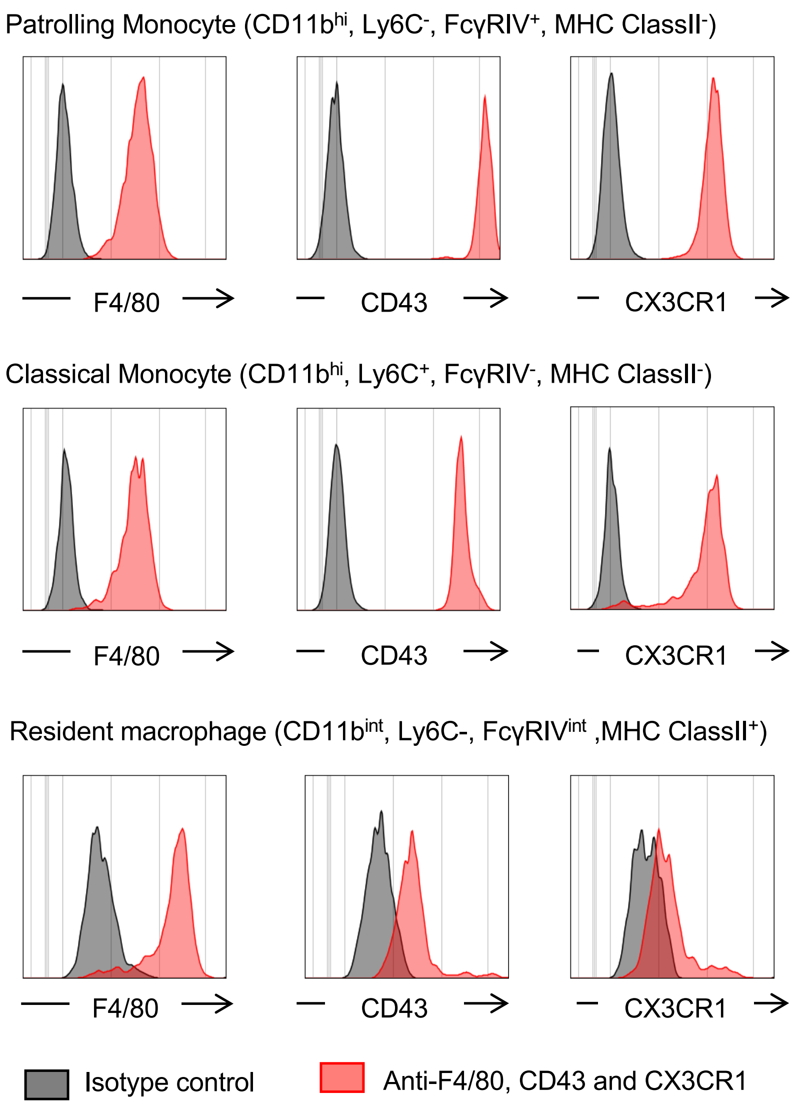

Supplementary Figure 3. Expression pattern of markers on monocyte subsets
Splenocytes were stained and gated by indicated markers. Expression of F4/80, CD43 and CX3CR1 on the subsets were shown as histograms.

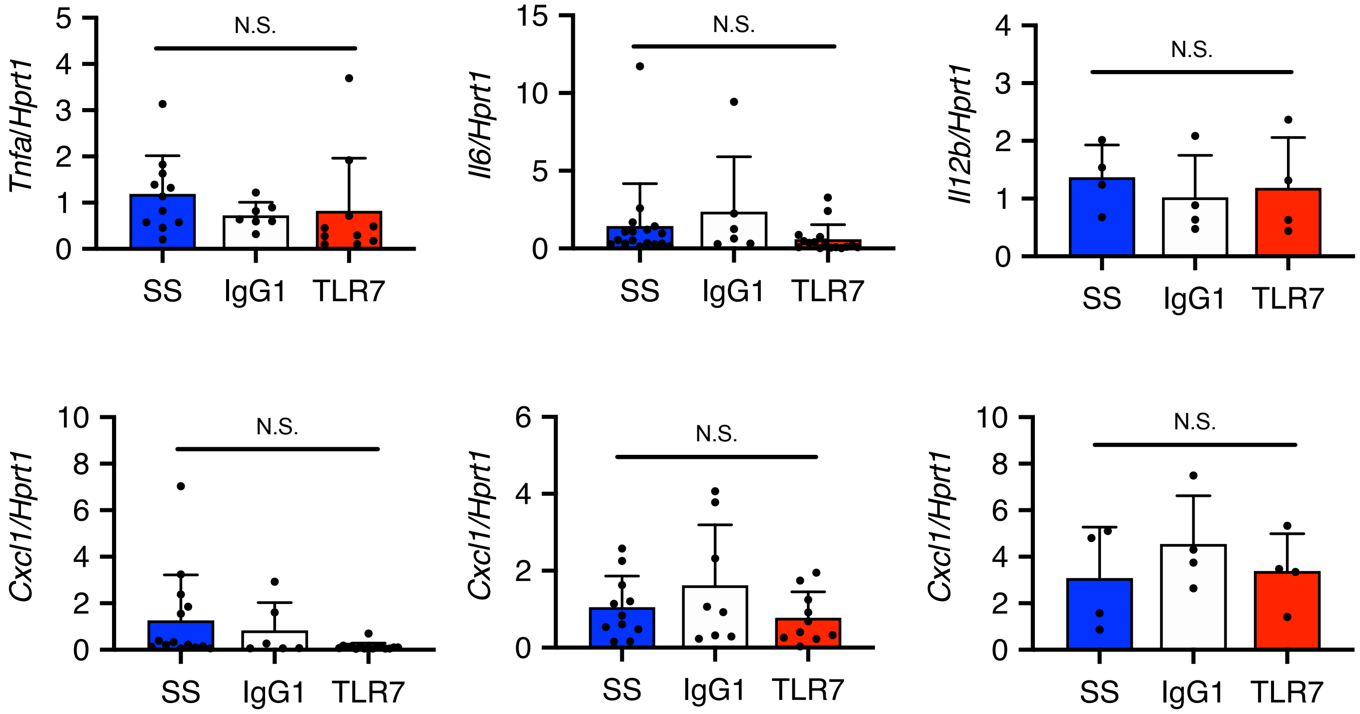

Supplementary Figure 4. Transcription of inflammatory cytokines in kidney were not changed by anti-TLR7

NZBWF1 mice were administered saline, IgG1 or anti-TLR7 mAb from ages of 12-16 to 35-40 weeks. Expression of mRNAs encoding indicated genes in kidney were analyzed by real-time PCR. Results are normalized by Hprl1 mRNA. Standard saline (SS, n ≥ 4), control IgG1 (IgG1, n ≥ 4), and anti-TLR7 mAb (TLR7, n ≥ 4).

Data were statistically analyzed using one-way ANOVA with Tukey's multiple comparison tests. N.S., not significant. Data are shown as individual points and as means ± SD for each experimental group.
